# Prognostication and risk factor stratification for survival of patients with hepatocellular carcinoma: a nationwide big data analysis

**DOI:** 10.1038/s41598-023-37277-9

**Published:** 2023-06-27

**Authors:** Jin Woo Choi, Soohee Kang, Juhee Lee, Yunhee Choi, Hyo-Cheol Kim, Jin Wook Chung

**Affiliations:** 1 Department of Radiology, Seoul National University Hospital, Seoul National University, 101 Daehak-Ro, Jongno-Gu, Seoul, 03080 Republic of Korea; 2 Medical Research Collaborating Center, Seoul National University Hospital, Seoul National University, Seoul, Republic of Korea

**Keywords:** Cancer therapy, Gastrointestinal cancer

## Abstract

This study was conducted to identify risk factors affecting overall survival (OS) and provide prognostication for patients with hepatocellular carcinoma (HCC) using nationwide big data. Between January 2008 and December 2014, 10,573 adult patients with new HCC were registered in a nationwide database. Among them, 6830 patients without missing data were analyzed to construct a prognostication system. A validation cohort of 4580 patients was obtained from a tertiary hospital. All patients were assumed to have received the best treatment. A conditional inference tree analysis was performed to establish a prognostic system. The C-index and calibration plot for 5-year survival were estimated for validation. As a result, the tumor burden (TB) grade was the most significant factor in determining OS, and the cutoff was TB3 (TB1‒3 versus TB4). The patients were ultimately divided into 13 prognosis groups. The C-indexes were 0.714 and 0.737 (95% confidence interval, 0.733–0.742) in the nationwide (derivation) and hospital (validation) cohorts, respectively. In the calibration plot, the 5-year survival of the validation cohort largely matched the 45-degree line. In conclusion, the proposed prognostication system with a simple tree structure enabled the detailed stratification of patient prognosis and visualized the strata of risk factors affecting OS.

## Introduction

Hepatocellular carcinoma (HCC) is one of the most complicated diseases due to its diverse clinical courses and inherently heterogeneous nature. Accordingly, there are more than 10 staging systems for HCC, and a myriad of studies have compared these systems^1–12^. Studies have inconsistently reported the superiority or inferiority of a specific system over others^13,14^. Among them, the Barcelona Clinic Liver Cancer (BCLC) system was initially derived from survival data of untreated and treated HCC patients, which facilitates a reliable prognostication and proposal of a treatment algorithm, and keeps updated as new evidence emerges^1,15,16^.

However, the selection of first-line treatment at each stage is frequently limited in real-world practice^17,18^. This is because curative treatments recommended by the system cannot be followed in some cases due to the patients’ comorbidities. Furthermore, it sometimes prevents some patients from surgical resection, although a potential survival gain is expected^19^. Patient heterogeneity within a BCLC stage has also been challenged^20,21^. The in-stage disparateness does not only induce deviation from the BCLC system but also precludes post-treatment comparisons of the different treatment options.

In this regard, BCLC prognostication is less valid in patients who are not managed with the first-line treatment. Risk factors affecting survival outcomes in patients managed using a personalized decision-making approach also need to be re-evaluated for prognostication. Moreover, given the wide uptake of personalized treatment for HCC in retrospective studies, it appears to be more reliable to analyze survival outcomes by assuming that every patient received the best treatment.

Given the diversity of HCC treatment modalities and the multifactorial nature of decision-making, it is challenging to propose a reliable prognostication system with a hospital-level cohort. Therefore, this study was conducted to identify the risk factors affecting overall survival (OS) and to provide detailed prognostication for patients with HCC using nationwide big data.

## Results

### Baseline characteristics

**Figure 1:**
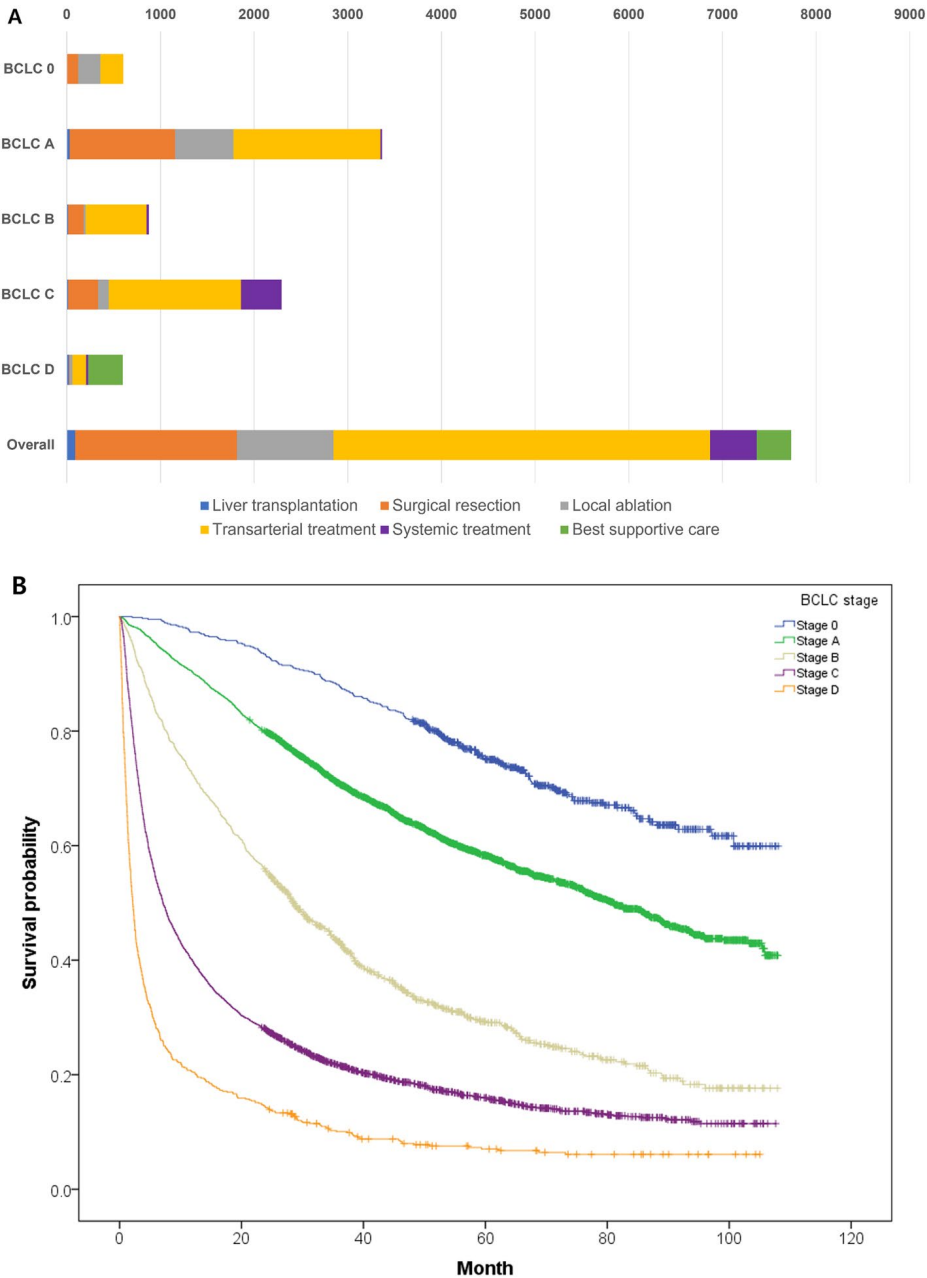
The initial treatment modality and overall survival depending on the Barcelona Clinic Liver Cancer (BCLC) stage. (**A**) Initial treatment modalities in each BCLC stage. (**B**) Overall survival of each BCLC stage.

The demographic data of the nationwide database are summarized in Table 1. The etiology of liver cirrhosis and hepatitis B and C viruses were identified in approximately 61.4% and 12.2% of the study population, respectively. Most variables had a trivial proportion of missing values, whereas the performance status was unknown in 11.1% of the total study population. According to the BCLC system, BCLC types A (38.8%, 3628/9358) and C (36.2%, 3387/9358) accounted for the majority of the patient population. Patients with BCLC stage 0 (6.7%, 623/9358) had a 5-year survival rate of 75.2%. The median survival rates were stratified from 81.1 months (95% confidence interval, 76.4–86.9) of BCLC A to 2.2 months (1.9–2.5 months) of BCLC D. First-line treatment was available for 7736 patients. Transarterial treatments were the most frequently used method across all BCLC stages, accounting for 52.0% (4023/7736) of the initial treatment (Fig. 1).

**Table 1:** Baseline characteristics of a nationwide database of hepatocellular carcinoma.

Variable	Value
Sex	
Male	7413 (79.2)
Female	1943 (20.8)
Unknown	2 (0.0)
Age (year)^†^	60.0 ± 11.4 (18‒113)
Enrollment year	
2008‒2010	4149 (44.3)
2011–2014	5209 (55.7)
Pathological diagnosis	
Yes, biopsy and/or surgery was performed	2811 (30.0)
No	6547 (70.0)
Smoking at the time of diagnosis	
Smoker	4213 (45.0)
Non-smoker	5049 (54.0)
Unknown	96 (0.1)
Diabetes Mellitus	
Yes	2265 (24.2)
No	7005 (74.9)
Unknown	88 (0.9)
Hepatitis B virus	
Yes	5750 (61.4)
No	3353 (35.8)
Unknown	255 (2.7)
Hepatitis C virus	
Yes	1131 (12.1)
No	7671 (82.0)
Unknown	556 (5.9)
Alcoholic liver cirrhosis	
Yes	3015 (32.2)
No	6172 (66.0)
Unknown	171 (1.8)
Performance status	
0	6581 (70.3)
1	1238 (13.2)
2	283 (3.0)
3	135 (1.4)
4	78 (0.8)
Unknown	1043 (11.1)
Child–Pugh class	
A	6702 (71.6)
B	2087 (22.3)
C	501 (5.4)
Unknown	68 (0.7)
Albumin-Bilirubin grade	
Grade 1	3761 (40.2)
Grade 2	4500 (48.1)
Grade 3	1054 (11.3)
Unknown	43 (0.5)
Model for End-stage liver disease score	
0‒10	6564 (70.1)
11‒20	2371 (25.3)
21‒30	220 (2.4)
> 31	41 (0.4)
Unknown	162 (1.7)
Largest tumor size (cm)	
≤ 2.0	2408 (25.7)
2.1‒5.0	3291 (35.2)
5.1‒10.0	1844 (19.7)
> 10.0	1000 (10.7)
Unknown	815 (8.7)
Tumor burden	
TB1	1439 (15.4)
TB2	3310 (35.4)
TB3	803 (8.6)
TB4	3773 (40.3)
Unknown	33 (0.4)
Vascular invasion	
Yes	2427 (25.9)
No	6931 (74.1)
Unknown	0 (0.0)
Extrahepatic spread	
Yes	1369 (14.6)
No	7989 (85.4)
Unknown	0 (0.0)
BCLC stage	
0	623 (6.7)
A	3628 (38.8)
B	1060 (11.3)
C	3387 (36.2)
D	660 (7.1)
Unknown	0 (0.0)
Alpha feto-protein	
< 200 ng/mL	5626 (60.1)
≥ 200 ng/mL	3136 (33.5)
Unknown	596 (6.4)

Numbers in parenthesis are percentages.

^†^Data are mean ± standard deviation (range).

### Risk factor stratification

**Figure 2:**
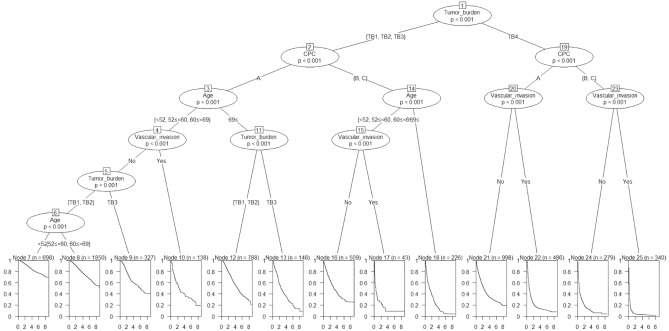
The conditional inference tree for prognostication of patients with hepatocellular carcinoma.

In total, 6830 patients were subjected to a conditional inference tree for risk factor stratification. Tumor burden (TB) was the most significant factor determining OS, and the cutoff was TB grade 3 (TB3) (TB grade 1–3 [TB1‒3] vs. TB grade 4 [TB4]). The second most significant factor was Child–Pugh class (CPC); thus, CPC A patients showed a significantly longer OS than CPC B or C patients in both TB groups (TB1‒3, TB4). Age (cutoff, 69 years) was the third most significant factor in patients with TB1‒3, whereas vascular invasion was the third most significant factor in patients with TB4. Patients were ultimately divided into 13 prognosis groups, depending on the risk factors for OS (Fig. 2).

### Validation of the prognostication system

**Figure 3:**
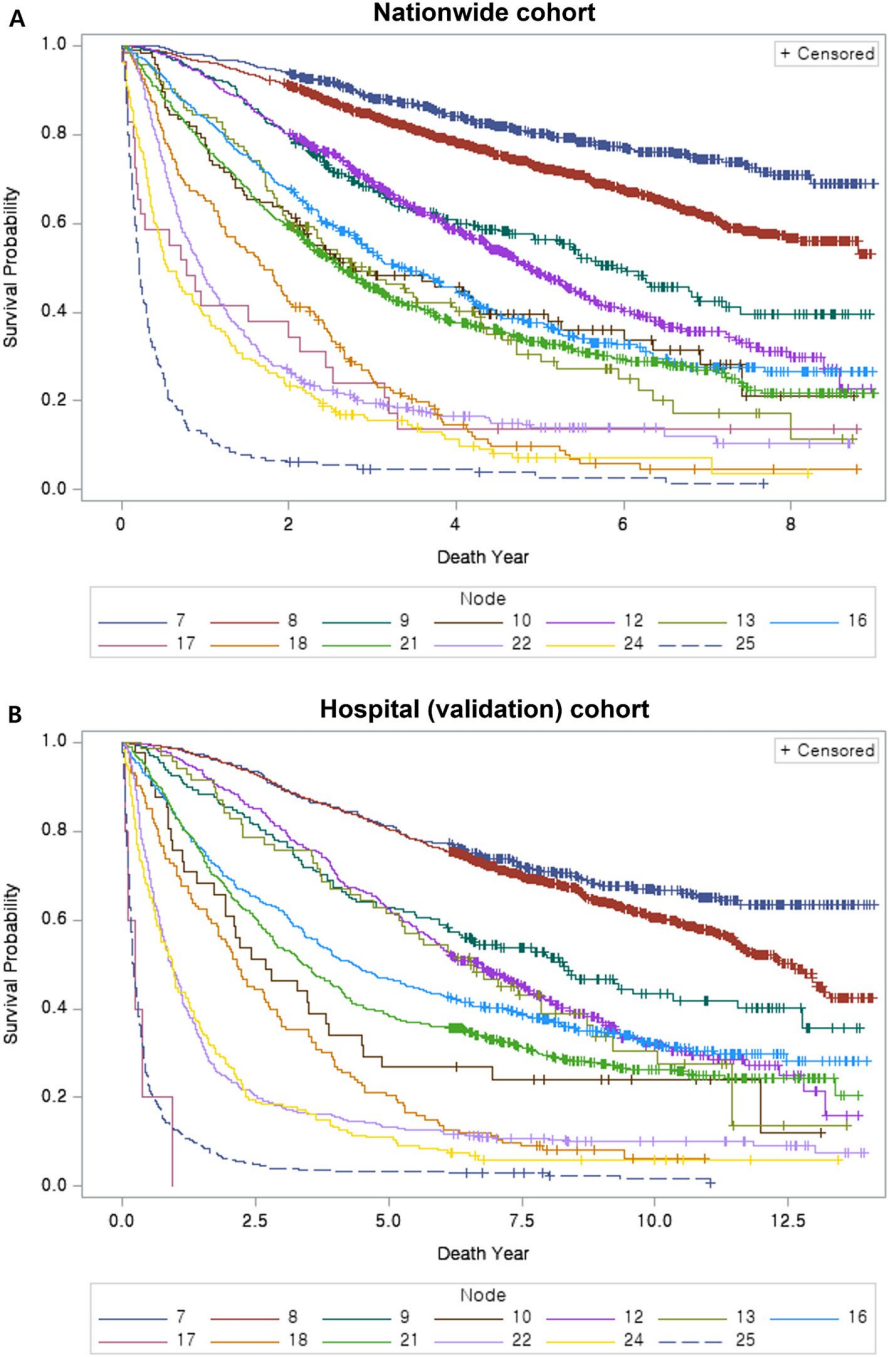
The overall survival of each prognostication group. (**A**) The Kaplan–Meier plot of the nationwide cohort. (**B**) The Kaplan–Meier plot of the hospital cohort for validation.

**Figure 4:**
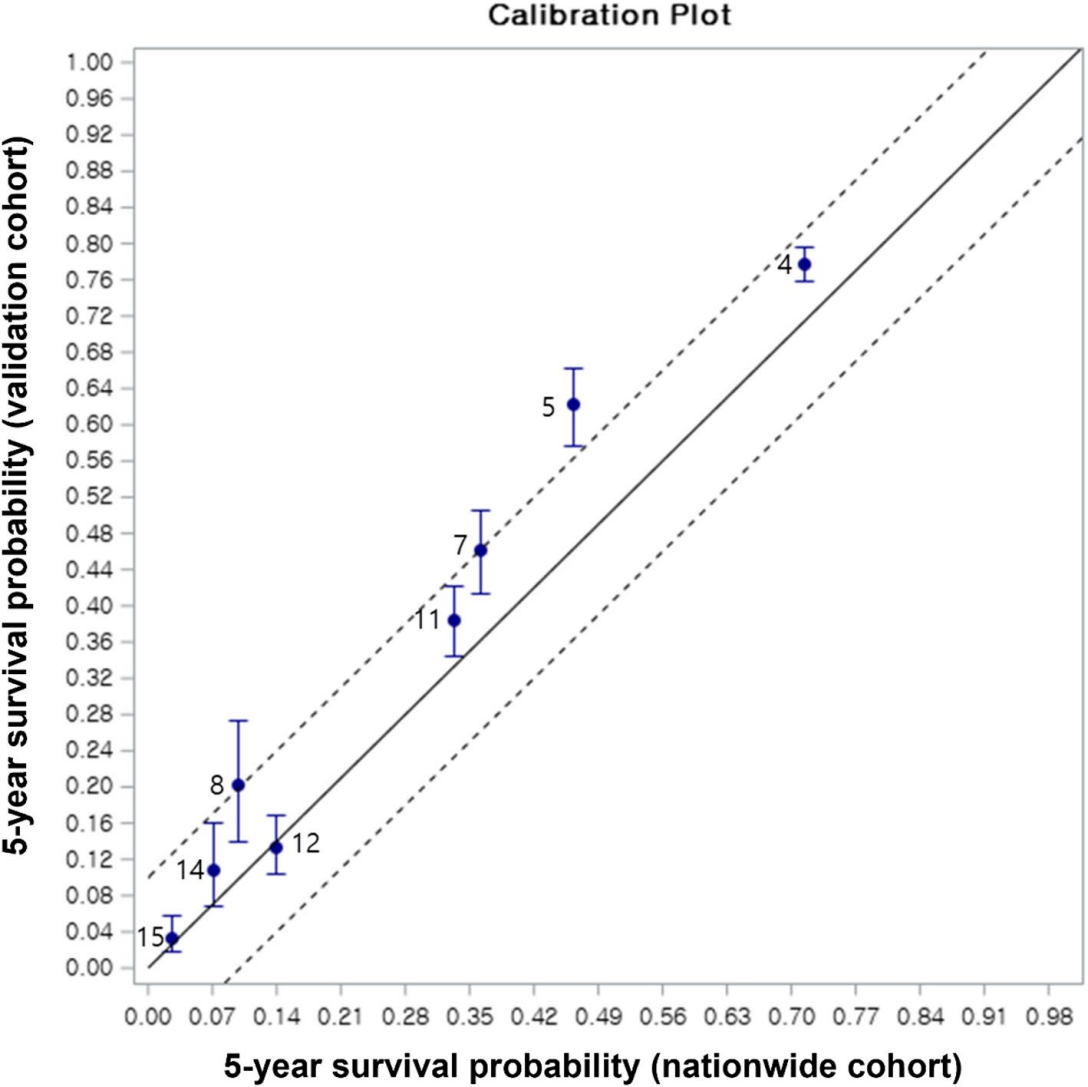
The calibration plots of observed (hospital cohort) versus predicted (nationwide cohort) overall survival at 5-year after diagnosis of hepatocellular carcinoma.

The cohort from a tertiary referral center was compared to the nationwide cohort, and significant differences in patient age (*p* < 0.001), etiology of liver cirrhosis (*p* < 0.001), CPC (*p* < 0.001), tumor burden (*p* < 0.001), and vascular invasion (*p* = 0.003) were noted on the chi-square or Wilcoxon rank-sum tests. However, the standardized difference was above 0.1 only in the etiology of liver cirrhosis (Table 2). Both cohorts showed similar trends in the 5-year survival rates for each node (Fig. 3). The concordance index (C-index) of the prognostication model was 0.714 (95% confidence interval, 0.707–0.719) and 0.737 (95% confidence interval, 0.733–0.742) in the nationwide (derivation) and hospital (validation) cohorts, respectively. The C-indexes of the BCLC system for the nationwide cohort and hospital cohort were found to be 0.646 (95% confidence interval, 0.641–0.651) and 0.703 (95% confidence interval, 0.707–0.699), respectively. These values were significantly inferior to those of the proposed system with *p*-values < 0.001 for both comparisons. In the calibration plot, the 5-year survival of the validation cohort largely matched the 45-degree line, but the validation cohort from a tertiary referral center tended to show better outcomes than those estimated based on the nationwide cohort-derived model (Fig. 4).

**Table 2:** Comparison of demographic data between the two cohorts.

	KLCA nationwide database (n = 6830)	Hospital cohort (n = 4580)	*p*-value	Standardized difference
Sex				
Male	5310 (77.8)	3589 (78.4)	0.435	0.015
Female	1520 (22.3)	991 (21.6)		− 0.015
Age (year)	60.28 ± 11.1	59.45 ± 10.49	< 0.001	− 0.077
Hepatitis B virus				
Yes	4191 (63.0)	3201 (69.9)	< 0.001	− 0.147
No	2466 (37.0)	1379 (30.1)		0.147
Hepatitis C virus				
Yes	879 (13.6)	511 (11.2)	< 0.001	0.076
No	5564 (86.4)	4069 (88.8)		− 0.076
Alcoholic liver cirrhosis				
Yes	2135 (31.8)	266 (5.8)	< 0.001	0.704
No	4589 (68.3)	4314 (94.2)		− 0.704
Child–Pugh class				
A	5433 (79.6)	3450 (75.6)	< 0.001	− 0.095
B	1244 (18.2)	964 (21.1)		0.073
C	153 (2.2)	151 (3.3)		0.065
Tumor burden				
TB1	1257 (18.4)	936 (20.4)	< 0.001	0.051
TB2	2831 (41.5)	1749 (38.2)		− 0.067
TB3	639 (9.4)	332 (7.3)		− 0.076
TB4	2103 (30.8)	1563 (34.1)		0.071
Vascular invasion				
Yes	1064 (15.6)	810 (17.7)	0.003	− 0.057
No	5766 (84.4)	3770 (82.3)		0.057
Alpha feto-protein (ng/mL)				
< 200 ng/mL	4795 (70.2)	3160 (69.0)	0.168	0.081
≥ 200 ng/mL	2035 (29.8)	1420 (31.0)

## Discussion

Owing to the frequent use of BCLC-discordant treatment options and the broad spectrum of OS in each stage, the prognostication part of the BCLC system makes little contribution to clinical practice. Given the aging trend in developed countries, suggesting an increase in patients with multiple comorbidities and uptake of less invasive surgical techniques, the discrepancy may become even larger in the future. In this context, this study established a conditional inference tree based on nationwide big data and validated by a large hospital cohort to present stratified risk factors and OS. Patients with HCC were divided into 13 different prognosis groups, and the strata of risk factors affecting OS were identified. Despite the complexity of the risk factor hierarchy and multiple prognosis groups, the simple tree structure provides an easy-to-follow system that allows users to identify the layers of risk factors at a glance. This study also assumed that every patient received best-fit treatment regarding the wide uptake of personalized approach in HCC management. Consequently, a practical and detailed prognostication system can be presented as a single figure.

Tumor burden was the most important risk factor in this study. HCC has a wide variety in terms of tumor size and multiplicity, resulting in the development of multiple staging systems for the tumor burden^22^. In line with the purpose of creating a practical prognostication system, the present study proposes a tumor burden classification method. As a result, the tumor burden appeared multiple times in the conditional inference tree, suggesting the usefulness of the tumor burden staging system. Although the Milan and up-to-seven criteria were originally proposed to select ideal candidates for LT^23,24^, they are also used to evaluate HCC patients managed by other treatments^22^. By adopting these criteria, the present study was able to build an easy-to-apply prognostication system.

Several retrospective studies have compared the survival outcomes of different treatment modalities. However, it is not within the scope of the present study to compare the effectiveness of each treatment modality. Because the determination of a treatment method, especially when a BCLC-discordant decision is made, reflects the underlying conditions that can potentially affect OS, it is not feasible to measure only the impact of a treatment, excluding the influence of underlying conditions. Although statistical methods, including propensity score analysis, can partially compensate for the effect of pretreatment conditions, they cannot consider factors that are not properly analyzed. For example, tumor location is one of the key factors determining the initial and subsequent treatment modalities as well as affecting tumor responses. HCC in the central liver or caudate lobe is likely to be treated using non-surgical methods, which may affect the OS of patients. The outcome of locoregional treatments is also substantially affected by tumor location and visibility on imaging^25^. However, there is no reliable tool that can measure tumor location-related treatment difficulty; thus, this factor cannot be compensated for in retrospective studies with ready-treated patients. Therefore, this study did not aim to compare the effect of each treatment method but rather to present the survival outcomes of patients managed by major treatment modalities.

This study had some limitations. Although the proportion of patients with missing data for each item was trivial, the fraction of patients with missing data were approximately a quarter of the nationwide cohort. This study only analyzed patients with a complete dataset, assuming that missing data occurred randomly. However, this approach may have introduced deviations in the OS measured in the present study from the actual outcomes. While the assumption that every patient received the best available treatment at the time of initial diagnosis aided in constructing a prognostication system that reflects real-world practices, it remains ambiguous whether all patients were indeed managed with the most ideal treatment methods. This question becomes particularly relevant given that the data were collected nationwide, and not solely from specialized referral centers. Although treatment crossover is very frequent during the management of patients with HCC^26^, only the initial treatment method was considered in this study. Hence, the proposed prognostication system cannot fully reflect the heterogeneity of survival outcomes, especially in patients with a low tumor burden. Although management tools for advanced HCC have become plentiful over the last decade, the present study did not reflect this trend. The prognosis of patients with advanced HCC was better than that presented in this study. Given that both the nationwide and hospital (validation) cohorts are derived from Korea, the generalizability of our results may be limited in populations outside Korea. The exclusion of patients who underwent liver transplantation (LT) and those with extrahepatic spread from the present study limits the applicability of our model to these specific patient populations.

In conclusion, the proposed prognostication system with a simple tree structure enabled the detailed stratification of patient prognosis and visualized the strata of risk factors affecting OS.

## Methods

### Nationwide patient cohort

**Figure 5:**
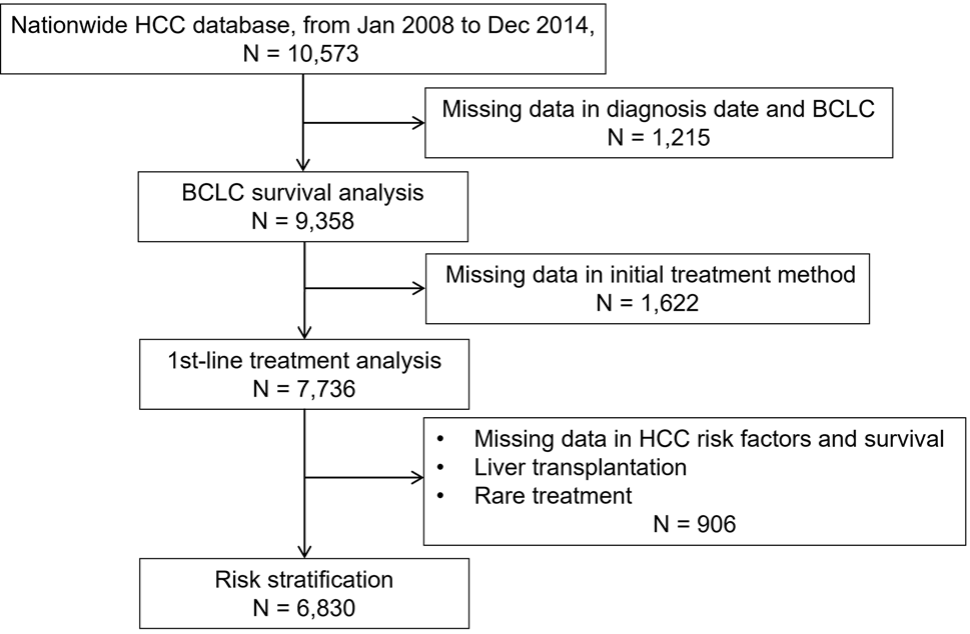
Flowchart of patient selection for each analysis.

This study was conducted in accordance with the ethical guidelines of the 1975 Declaration of Helsinki. The Institutional Review Board of Seoul National University Hospital approved this study and waived the requirement of informed consent due to the retrospective study nature. The Korean Liver Cancer Association randomly sampled approximately 10% of newly diagnosed HCC patients from the Korea Central Cancer Registry every year for academic research purposes and archived their demographic data, underlying liver disease, performance status, laboratory findings, imaging findings at the initial presentation, and the first treatment method after anonymization. Diagnosis of HCC was primarily made based on typical imaging patterns observed on dynamic CT or MRI, or through biopsy. The survival/death information was updated annually based on data from the Ministry of Interior and Safety of Korea. Between January 2008 and December 2014, 10,573 adult patients with new HCC were registered in the Korean Liver Cancer Association nationwide database, accounting for 9.4% of all new patients in Korea. As this is a retrospective registry, some data were missing because these were not included in the patients’ medical records. Therefore, this study initially selected 9358 patients with complete registration, in terms of the date of diagnosis and BCLC stage (Fig. 5).

### Patient selection for risk factor analysis

To identify and stratify the risk factors, this study selected patients with essential data for decision-making. Patients with missing data on sex, age, performance status, CPC, largest tumor size, tumor number, vascular invasion on dynamic computed tomography or magnetic resonance imaging, extrahepatic spread, serum alpha-fetoprotein level, initial treatment method, or survival data were excluded. LT is a viable option for selected patients across the BCLC stages, and it substantially alters patients’ clinical course. Therefore, patients who underwent LT as the first treatment were excluded from analyses of risk factor stratification for a survival outcome. Patients with extrahepatic spread were also excluded because imaging studies for determining extrahepatic spread were not performed consistently during the registration period. Therefore, 6830 patients were analyzed to identify factors affecting OS.

### Risk factors

Patient sex, age, performance status, CPC, tumor burden, vascular invasion, and alpha-fetoprotein were regarded as potential risk factors affecting OS. Age was divided into quartiles to create four age strata. Given the multicentricity of HCC, tumor burden was determined by considering the largest tumor size and the number of tumors as follows: TB1, single tumor < 2 cm; TB2, beyond TB1 and within the Milan criteria^23^; TB3, beyond TB2 and within the up-to-seven criteria^24^; and TB4, beyond the up-to-seven criteria. TB and vascular invasion were judged based on pretreatment computed tomography or magnetic resonance imaging, while pathological findings after surgery were not reflected in the classification.

### Treatment

Given that this nationwide retrospective registry consisted of data spanning seven years, diverse treatment modalities were recorded for the patients. This study simplified the treatment methods into four categories: (1) surgical resection, (2) local ablation (e.g., radiofrequency ablation, percutaneous ethanol injection), (3) transarterial therapy (e.g., transarterial embolization, transarterial chemoembolization [conventional and drug-eluting embolic], transarterial chemoinfusion, radioembolization), (4) systemic therapy (e.g., sorafenib, systemic chemotherapy) or best supportive care. Patients managed with systemic therapy and best supportive care were grouped because sorafenib was not widely used for patients without extrahepatic spread during the patient enrollment period in Korea (1.7%, 117/6830), and only a small portion of patients was managed with systemic therapy or best supportive care (3.6%, 243/6830). Given the wide uptake of personalized treatment for HCC, all patients in this study were considered to have received the best treatment possible.

### Hospital patient cohort

A hospital patient cohort obtained from a tertiary referral center was used to validate the proposed prognostication system based on a nationwide database. The cohort consisted of 4580 consecutive patients who were first diagnosed with HCC at the hospital between January 2005 and December 2012. The potential risk factors were tabulated after a review of electronic medical records, and survival data were acquired from the same source in the nationwide database, the Ministry of Interior and Safety of Korea.

### Statistical analysis

Patients in the nationwide database were divided according to their BCLC stage, and their OS was evaluated using the Kaplan–Meier method and log-rank test. The survival time was estimated from the time of first treatment to the time of death. Conditional inference tree analysis was conducted to classify patients according to the risk factors for OS^27^. The patients were initially split into two subsets as determined by a specific cutoff value that made the most different survival between the two subsets. In each subset, the split process continued until the survival of the next two subsets was not statistically significant. Because of the large nationwide data, the tree size could be inefficiently large by overpowered tests for split. Therefore, tree size was controlled by determining the level of statistical significance as 0.001. Survival for each group was estimated based on the results of the conditional inference tree.

In the validation hospital cohort, the patients’ baseline characteristics were compared to those of the nationwide database using the chi-square test or Wilcoxon rank-sum test. To supplement overpowered tests that can potentially inflate inter-group differences, a standardized difference, the difference of two means (or fractions) divided by the difference of standard deviations, was calculated, and an absolute value < 0.1 was regarded as supporting the assumption of balance between the two groups^28,29^. The validation data were grouped according to the conditional inference tree generated using nationwide data, and Uno’s C-index was estimated to evaluate the discrimination performance of the tree in both cohorts. The survival at 5 years in each group was estimated and compared with expectations using a calibration plot.

Statistical analyses were conducted using SAS software (SAS, Version 9.4, SAS Institute, Cary, North Carolina, United States) and R software (R for Windows, version 4.0.5/R package—party, R Foundation for Statistical Computing, Vienna, Austria). All statistical tests, except for the conditional inference tree, were two-sided at a 5% level of significance.

## Data Availability

The data that support the findings of this study are not publicly available due to the Personal Information Protection Act, but are available from the corresponding author upon reasonable request.
